# Cloning, heterologous expression, and expression analysis of *SinSyn7* gene from *Sinomenium acutum*

**DOI:** 10.1371/journal.pone.0327959

**Published:** 2025-07-09

**Authors:** Jiabei Chen, Xinyu Chen, Li Zhang, Shiyi Huang, Xinbo Chen, Xueshuang Huang, Hua Yang

**Affiliations:** 1 College of Bioscience and Biotechnology, Hunan Agricultural University, Changsha, China; 2 Yuelushan Laborary, Changsha, China; 3 Hunan Provincial Key Laboratory for Synthetic Biology of Traditional Chinese Medicine, Hunan University of Medicine, Huaihua, China; Federal University of Espirito Santo, BRAZIL

## Abstract

The main component of *Sinomenium acutum*, sinomenine, has anti-inflammatory, analgesic, and immunosuppressive effects. In order to achieve the biosynthesis of sinomenine, the synthase gene *SinSyn7* was cloned from *Sinomenium acutum* and expressed heterologously in brewing yeast WAT11, and its bioinformatics analysis and tissue-specific expression were investigated. The results showed that the coding region (CDS) of *SinSyn7* is 1602 bp, encoding 523 amino acids, with an isoelectric point pI of 7.28. The *SinSyn7* protein is a hydrophilic protein, without signal peptides, and has a transmembrane domain. It belongs to the cytochrome P450 superfamily and is mainly composed of α-helices and irregular coils to form a secondary structure. The molecular docking results showed that the binding free energies of the six ligands to *SinSyn7* ranged from −9.0 to −7.3 kcal· mol^-1^, and all exhibited strong binding abilities. Alanine scanning and saturation mutagenesis analysis revealed that there are 5 key amino acid residues involved in the *SinSyn7* catalyzed (*S*)-reticuline sophocarpine reaction. The content trends of sinoacutine and sinomenine in different tissues of *Sinomenium acutum* were consistent. The qRT-PCR results showed that the expression level of the *SinSyn7* gene was relatively high in the rhizome of *Sinomenium acutum*. This study provides insights into further revealing the role of *SinSyn7*.

## Introduction

*Sinomenium acutum* is the dry stem of *Sinomenium acutum* (Thunb.) Rehd. et Wils. var. *cinereum* Rehd. Et Wils. and *Sinomenium acutum* (Thunb.) Rehd. et Wils.), and it has been demonstrated to be effective in treating joint swelling, edema, and athlete’s foot [1]. Wang et al.’s [2] study indicated that, apart from some national nature reserves, the resources of *Sinomenium acutum* in many regions of China had been destroyed, with the species even on the brink of extinction in certain areas. According to statistics, there are more than 250 active components in *Sinomenium acutum*, which are essential for various health care products. The main active components of *Sinomenium acutum* are alkaloids such as morphinanes, isoquinolines, and apophines [3]. Sinomenine is an isoquinoline alkaloid extracted from *Sinomenium acutum*, which accounts for 80% of the total alkaloids in *Sinomenium acutum* [4]. Sinomenine has high anti-inflammatory and immunosuppressive effects, and has been widely used in the clinical treatment of autoimmune diseases such as rheumatoid arthritis [5–7]. Sinoacutine is a morphinane alkaloid, which is similar in structure to sinomenine. He et al. [8] found that sinomenine was transformed by sinoacutine. *SinSyn7* gene is the homologous gene of sinoacutine synthetase, but sinoacutine synthetase has low catalytic activity. Therefore, the coding sequence of the *SinSyn7* gene was obtained, and then its possible catalytic ability and the correlation of its expression with sinoacutine content in different tissues of *Sinomenium acutum* plants were analyzed, which could provide a reference for finding sinoacutine synthase with better catalytic activity, and also lay a foundation for subsequent analysis of sinomenine synthesis pathway.

To date, 91 alkaloids have been identified in *Sinoimenicum acutum*, with isoquinolines, aporphines, morphines, being the predominant structural types. [9]. As for the biosynthesis pathway of isoquinoline alkaloids, the precursor substance tyrosine was catalyzed by a series of enzymes to produce an important intermediate product (*S*)-reticuline, which can form all types of isoquinoline alkaloids through methylation, demethylation, isomerization, and C-C phenylphenol coupling [10–13]. In the downstream synthesis pathway of morphinane, (*S*)-reticuline was isomerized to (*R*)-reticuline, which was then subjected to a series of enzyme-catalyzed reactions to produce morphine [14–18]. Drugs with different chiral structures exhibited different or even opposite effects [19]. Studies showed that (*S*)-reticuline can be catalyzed by the SinSyn enzyme to produce sinoacutine, which was then subjected to a series of enzymatic reactions to produce sinomenine [20–22]. SinSyn enzymes belonged to the cytochrome P450 superfamily, and the yeast strain *Saccharomyces cerevisiae* WAT11 was originally engineered to overexpressed Arabidopsis P450 reductase [23] and was identified as a good heterologous host for P450 protein expression in plants [24–26]. So far, it has been known that sinomenine, as the active component for the treatment of rheumatoid arthritis is rich in *Sinomenium acutum*; however, the complete pathway of sinomenine biosynthesis has not yet been clarified, the catalytic activity of sinoacutine synthase SinSyn is low, and the distribution of sinoacutine in different tissues of *Sinomenium acutum* and related reasons have not been reported.

This study employed a multifaceted approach, including gene cloning, bioinformatics analysis, heterologous expression in the WAT11 system, and expression analysis, to comprehensively elucidate the molecular characteristics and potential catalytic activity of the *SinSyn7* enzyme. Furthermore, the key sites of the *SinSyn7* protein, as well as the site mutation design that may improve the affinity between *SinSyn7* enzyme and (*S*)-reticuline, were predicted, so as to provide a basis for further functional identification of the *SinSyn7* gene, provide a theoretical basis for the screening of sinoacutine synthase with stronger activity, and lay a foundation for studying the anabolic mechanism of sinomenine.

## Materials and methods

The study was conducted at the experimental base of Hunan Zhenqing Pharmaceutical Group Co., Ltd. and the College of Bioscience and Technology, Hunan Agricultural University, from March 2022 to June 2024.

### Experimental materials

#### Plant materials.

The roots, stems, and leaves of No. 2, No. 13, No. 28, and No. 33 *Sinomenium acutum* plants were collected from the experimental base of Hunan Zhengqing Pharmaceutical Group Co., Ltd.

#### Strains and vectors.

*Escherichia coli* Top 10 competent cells (AngYuBio Shanghai). pEASY-T1 Cloning Vector (TransGen Biotech, Beijing).

### Reagents

Plant Total RNA Isolation Kit and HiScript-TS 5’/3’ RACE Kit (Vazyme, Nanjing); DNA Gel Rapid Purification Kit, pEASY-T1® Cloning Kit, DL2000 DNA Maker, *Blue Plus*® Protein Maker (14–100 kDa), *EasySee*® Western Maker (25–90 kDa) and Plasmid Mini Kit (TransGen, Beijing); Blunt-End DNA A Tailing Kit (Solarbio, Beijing). 12.5% PAGE Gel Rapid Preparation Kit and Tris-glycine-SDS Electrophoresis Buffer (Epizyme Biomedical, Shanghai), Coomassie Brilliant Blue Staining Solution, Destaining Solution, RAPI Lysate and PMSF Protease Inhibitor (Servicebio, Wuhan), His-Tag Mouse Monoclonal Antibody (HRP Conjugated) (Beyotime, Shanghai); Highly sensitive ECL luminescence reagent (Sangon Shanghai). 5 × SDS-PAGE Protein Loading Buffer (Boster, Wuhan).

### Cloning of *SinSyn7* gene

#### Total RNA isolation and testing.

RNA was isolated from roots, stems, and leaves from *Sinomenium acutum* using the Plant Total RNA Isolation Kit (Vazyme). 5 μL of RNA samples were taken, and 1 μL of 6 × loading buffer was added. Samples were applied on 1% agarose gel and electrophoresis was performed at 180 V for 10 min to measure the RNA integrity. 1 μL of RNA sample was pipetted to measure the concentration, OD260/280 and OD260/230 values of RNA sample by microvolume spectrophotometer.

#### Cloning of *SinSyn7* gene.

[1] Primer design: The gene-specific primer (GSP) of the *SinSyn7* gene was designed using the SnapGene 6.0.2 software. The primer was designed as close to the cDNA end as possible. The specific primer sequences are shown in (S1 Table).
[2] First-strand cDNA synthesis. Reverse transcription was performed according to the instructions of the HiScript-TS 5’/3’ RACE Kit.

#### Bioinformatics analysis of *SinSyn7* gene.

The obtained 5’RACE sequence was spliced with the known sequence using SnapGene software to obtain the correct CDS of sinoacutine synthase *SinSyn7* gene. Predictive analyses were performed for the amino acid sequence (by Translate, https://web.expasy.org/translate/), physicochemical properties (by EXPASy, https://web.expasy.org/protparam/), hydrophilicity and hydrophobicity (by ProtScale, https://web.expasy.org/protscale/), signal peptide (by SignalP4.1, https://services.healthtech.dtu.dk/services/SignalP-4.1/), subcellular location (by WoLF PSORT, https://wolfpsort.hgc.jp/), transmembrane domain (by TMHMM2.0, http://www.cbs.dtu.dk/ services/TMHMM-2.0/), conserved domain (by CDD, https://www.ncbi.nlm.nih.gov/Structure/cdd/wrpsb.cgi), secondary structure (by SOPMA, https://npsa-pbil.ibcp.fr/cgi-bin/npsa_automat.pl?page=npsa_sopma.html and ESPript, https://espript.ibcp.fr/ESPript/cgi-bin/ESPript.cgi), and protein function (by KEGG, https://www.kegg.jp/) of the *SinSyn7* gene. *SinSyn7* protein modeling was performed using the AlphaFold2 online platform (https://colab.research.google.com/github/sokrypton/ColabFold/blob/main/AlphaFold2.ipynb). A phylogenetic tree was drawn using MEGA.X.

The ligand small molecules were downloaded from PubChem (https://pubchem.ncbi.nlm.nih.gov/) and converted to PDB format using OpenBabel 2.4.1 software. PyMol software was used to hydrogenate and dehydrate the modeled proteins. Using AutoDock Vina molecular docking, the complexes with the lowest scores were outputted by PyMol, and 3D and 2D maps of the complex interactions were visualized and plotted using the Discovery Studio 2019 Client (DS2019) software. The results of molecular docking were studied for virtual amino acid mutations using the Discovery Studio 2019 Client (DS2019) software [27]. Heme was removed with PyMol. Using Calculate Mutation Energy (Binding) in DS2019, a 10Å alanine scan, and a 4Å saturation mutation scan were performed for amino acid residues involved in protein molecular docking. If the mutation energy was −0.5 to 0.5 kcal·mol^-1^, the effect was considered neutral, indicating that the mutation had no effect on affinity; if the mutation energy was greater than 0.5 kcal·mol^-1^, the effect was considered destabilizing, indicating that the mutation would reduce the affinity and weaken the interactions; if the mutation energy was less than −0.5 kcal·mol^-1^, the effect was considered stabilizing, indicating that the mutation would increase the affinity and enhance the interactions.

### Yeast expression and protein purification

The identified coding sequence of the *SinSyn7* gene was synthesized at Tsingke Biotech, and the expression vector pESC-his-*SinSyn7* of *Saccharomyces cerevisiae* was successfully constructed. The pESC-His-*SinSyn7* plasmid extracted from *Escherichia coli* Top10 was transformed into the *Saccharomyces cerevisiae* WAT11 using an appropriate kit, and the WAT11 that failed in induced expression (pESC-His-*SinSyn7*) was used as a control. The single colony in the SC-His medium plate was inoculated in 1 mL of SC-His liquid medium containing 2% glucose for growth at 30 °C for 24 h. Colony PCR with GAL10 primer was performed to identify positive clones. 100 μL of positive yeast solution was inoculated into 10 mL of SC-His glucose liquid medium, and then incubated on a shaker at 30 °C and 200 rpm for 36–48 h. The cells were centrifuged at 6000 rpm for 3 min and then resuspended with 10 mL of sterile water at 6000 rpm for 3 min, and the supernatant was discarded; a total of 3 runs were performed. The cells were resuspended with 100 mL of 2% galactose SC-his liquid medium and induced (at 30 °C and 200 rpm for 2–3 days of growth). The cells were centrifuged at 6000 rpm for 3 min, and the supernatant and pellets from the culture medium were collected. The pellets were resuspended with 10 mL PBST for 3 runs; 10 mL of PBST and 100 μL PMSF protease inhibitor were added, and the cells were well mixed by resuspension. The supernatant of the homogenized pellet solution was obtained after ultrasonic homogenization (at 55W and 6 s on/4 s off, for 30 min) and centrifugation at 4 °C and 10000 rpm for 10 min. 40 μL each of uninduced supernatant, induced supernatant, uninduced pellets (supernatant of homogenized pellet solution), and induced pellets (supernatant of homogenized pellet solution) was taken, and 10 μL of 5 × loading buffer was added and mixed well; the sample was processed at 100 °C for 5 min, and 20 μL of the sample was taken for SDS-PAGE and Western blot to analyze the protein expression.

1 mL of positive yeast solution was inoculated into 100 mL of SC-his glucose liquid medium, and then incubated on a shaker at 30 °C and 200 rpm for 36–48 h. After collecting and washing the cells following the steps above, the cells were transferred to 1 L of 2% galactose SC-his liquid medium for resuspension and induction (at 30 °C and 200 rpm for 2–3 days of growth). After the induction was completed, the cell pellets were collected and centrifuged at 7000 rpm for 10 min; then, 10 mL of lysis buffer (5 mM imidazole) was added and resuspended; the PMSF protease inhibitor was added and resuspended to mix the cells well. After ultrasonic homogenization (at 55 W and 6 s on/4 s off for 30 min), the cells were centrifuged at 4 °C and 10000 rpm for 10 min to separate the cell supernatant and pellets, and the cell supernatant was separated and purified with Ni-NTA packing. The purified protein was analyzed using SDS-PAGE and Western Blot.

### Assay of the sinoacutine content in *Sinomenium acutum*

Sinoacutine was extracted using the method of *Chinese Pharmacopoeia*. The sinoacutine reference standard was used to prepare the solutions at the concentrations of 10 μg·mL^-1^, 50 μg·mL^-1^, 100 μg·mL^-1^, 140 μg·mL^-1^, and 200 μg·mL^-1^, respectively, with methanol as the vehicle. The solution at each concentration level was filtered to a liquid phase vial using 0.22 μm organic filter membrane. 5 μL of the resultant solution was taken for HPLC.

### Expression analysis of *SinSyn7* gene

Sequence-specific primers for the *SinSyn7* gene, *SinSyn7*-F and *SinSyn7*-R, were designed by Oligo7 software (S1 Table). With U6 used as the internal reference gene, the relative expression of the *SinSyn7* gene in different tissues of several *Sinomenium acutum* plants was determined. To this end, the two-step PCR procedure was used, and the ΔΔCT method was used for the results. For the comparison among different tissues of the same plant and the comparison of the same tissue among different plants, the expression in the leaves of the No. 2 plant was used as the control, and the relative expression of the *SinSyn7* gene was calculated according to 2-ΔΔCT.

### Correlation and data analysis

Pearson correlation analysis was performed to investigate the correlation of the sinoacutine content in different tissues of *Sinomenium acutum* plants with the *SinSyn7* expression. The experimental data were processed by WPS Office, the ANOVA analysis and multiple comparisons were performed using GraphPad Prism 9.5.0 software, the experimental results were expressed as mean ± standard deviation (mean ± SD, n = 3), and two-way ANOVA was used for significance test.

## Results

### Total RNA isolation results

The total RNA electrophoresis results were observed in a gel imaging system (Shenhua Science Technology Co., Ltd., Hangzhou), and the integrity of the RNA samples was good. Based on the OD values of RNA samples of No. 2, No. 12, No. 28, and No. 33 plants, the RNA of the root of No. 2 plant with the best quality was selected as the template for 5’RACE amplification.

### Cloning of *SinSyn7* gene

**Fig 1:**
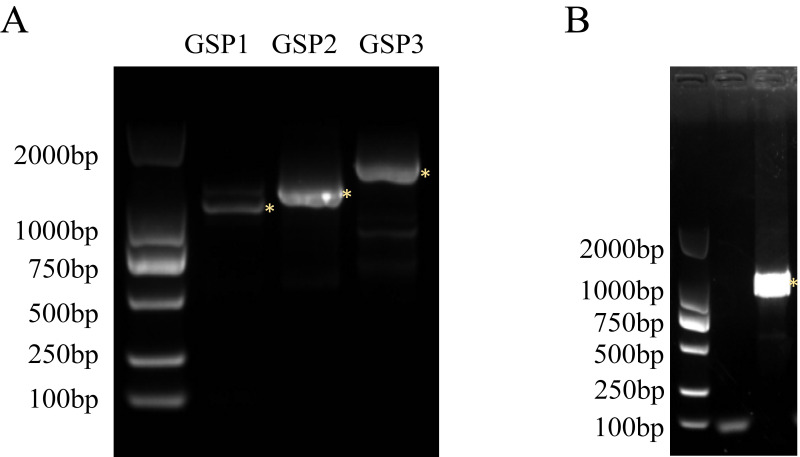
PCR amplification of *SinSyn7.* A. 5′ RACE amplified product (yellow asterisk indicates the correct amplification band); B. PCR amplification product of CDS of colony *SinSyn7*.

According to the known sequence of the *SinSyn7* gene in the whole genome of *Sinomenium acutum*, three 5’RACE primers were designed by SnapGene for 2 runs of PCR amplification, and 3 fragments were obtained, with the length being approx. 1500 bp, 1600 bp, and 1800 bp, respectively (**Fig 1**). The GSP1 primer was selected for extensive PCR amplification. The CDS length of the complete *SinSyn7* gene obtained by splicing the 5’RACE sequencing results with the known sequence of *SinSyn7* was 1602 bp (**Fig 1**).

### Bioinformatics analysis of *SinSyn7* gene

#### Basic bioinformatics analysis of *SinSyn7* protein.

**Fig 2:**
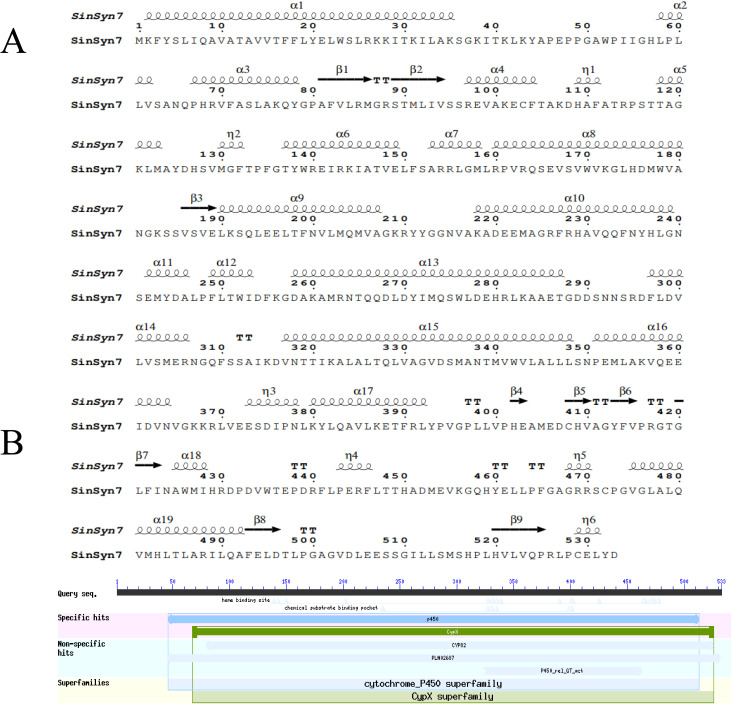
Prediction of the secondary structure of *SinSyn7* protein by ESPript and Conserved domain of *SinSyn7* protein.

**Fig 3:**
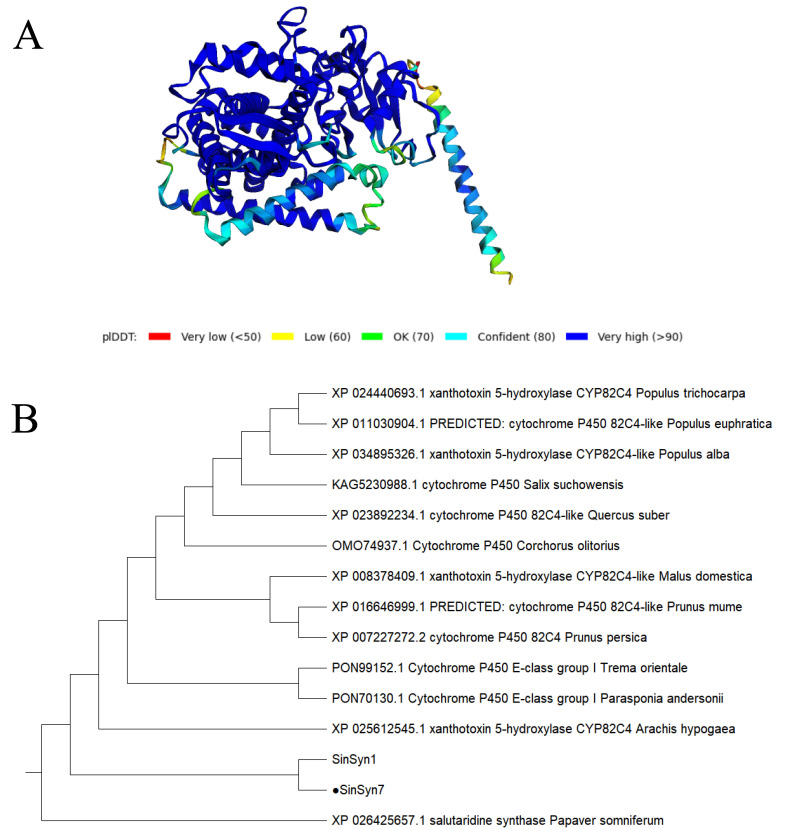
Tertiary structure of *SinSyn7* protein and Amino acid homology analysis of *SinSyn7* protein.

The prediction analysis for the physicochemical properties of *SinSyn7* protein showed that the molecular weight of *SinSyn7* protein was 59.98 kDa, the number of amino acids was 533, the theoretical isoelectric point PI was 7.28, the molecular formula was C2709H4248N726O760S26, the instability coefficient was 40.21, and the mean hydrophilicity was −0.071. On the whole, the *SinSyn7* protein was unstable and hydrophilic. There was no signal peptide in the amino acid sequence of *SinSyn7* protein; therefore, *SinSyn7* protein was considered a non-secreted protein. The amino acids 1–2 of the *SinSyn7* protein were intracellular, and amino acids 26–355 were extracellular; therefore, the *SinSyn7* protein had a transmembrane domain. By predicting the conserved domain of *SinSyn7* protein, *SinSyn7* protein was found to belong to the cytochrome P450 superfamily, and contained heme binding sites within the conserved domain (**Fig 2**). Subcellular location results showed that *SinSyn7* protein exhibited the highest score of 10 points in chloroplasts, followed by extracellular (2 points), nucleus (1 point), and vacuole (1 point). The analysis of the secondary structure of *SinSyn7* protein showed that α-helixes, random coils, extended chains, and β turns accounted for 46.90%, 36.96%, 36.96%, and 4.50%, respectively, suggesting that the secondary structure was dominated by α-helixes, followed by random coils (**Fig 2**). The tertiary structure model of the *SinSyn7* protein was predicted using AlphaFold2, as shown in **Fig 3**. The *SinSyn7* protein was most closely related to the homologous enzyme SinSyn1, followed by *Papaver somniferum* (XP 026425657.1) synthetase (**Fig 3**).

#### Docking of *SinSyn7* protein with ligand molecules.

In order to preliminarily investigate whether both *SinSyn7* protein and its homologous protein SinSyn1 could catalyze (*S*)-reticuline to produce sinoacutine [20], (*S*)-reticuline and sinoacutine were selected as ligands. According to literature, there were several isomers of sinoacutine, i.e., corytuberine and (*S*)-scoulerine, which were also used as ligands [28,29]. (*R*)-reticuline was a chiral symmetric isomer of (*S*)-reticuline, and thereby was also used as a ligand for study [17]. (*R*)-scoulerine was a chiral symmetric compound of (*S*)-scoulerine, and thereby was also used as a docking ligand. Therefore, a total of 6 ligand molecules were used in this study, among which (*S*)-reticuline and (*R*)-reticuline were substrate ligands, while the sinoacutine, corytuberine, (*R*)-scoulerine, and (*S*)-scoulerine were possible product ligands. After *SinSyn7* protein was docked with the above 6 ligand molecules, it was found that the binding of the 6 ligands to *SinSyn7* protein was located in the groove formed by the random coils between the α-10 helix (Lsy218–Asn239) and the α-16 helix (Asn350–Asn364) at the binding cavity mouth.

**Fig 4:**
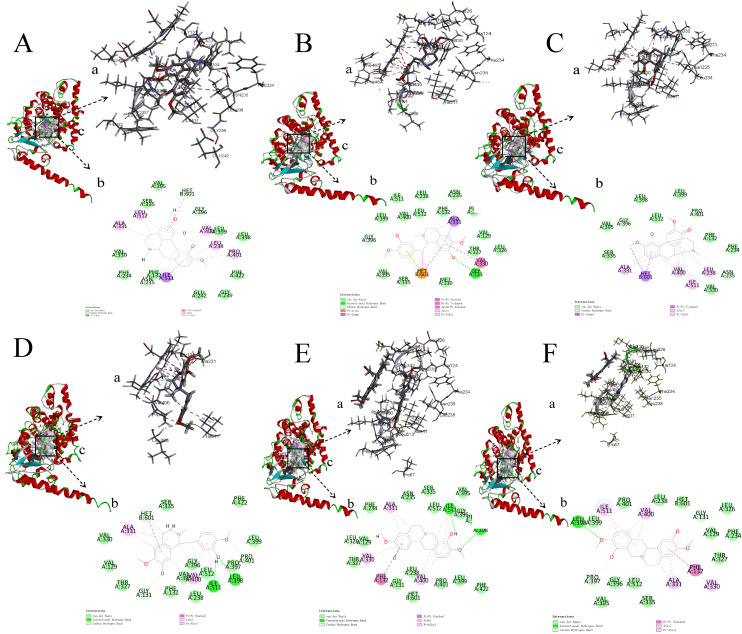
Binding modes of corytuberine, *(R)*-reticuline, sinoacutine, *(S)*-reticuline, *(S)*-scoulerine and *(R)*-scoulerine to the protein terminal pocket of *SinSyn7.* A. Binding mode of corytuberine to protein terminal pocket of *SinSyn7*; B. Binding mode of *(R)*-reticuline to protein terminal pocket of *SinSyn7*; C. Binding mode of sinoacutine to protein terminal pocket of *SinSyn7*; D. Binding mode of *(S)*-reticuline to protein terminal pocket of *SinSyn7*; E. Binding mode of *(S)*-scoulerine to protein terminal pocket of *SinSyn7*; F. Binding mode of *(R)*-scoulerine to protein terminal pocket of *SinSyn7*. a: Interaction; b: 2D binding mode; c: binding pocket; the same below.

The binding free energy of the ligands to *SinSyn7* protein was between −9.0 and −7.3 kcal·mol^-1^ (S2 Table). To be specific, the lowest binding free energy to *SinSyn7* (−9.7 kcal·mol^-1^) was found in corytuberine; the amino acid residues for hydrophobic interaction were Phe132, Val330, Val400, and Ile511, and no hydrogen bond was formed (Fig 4A). A lower binding free energy to *SinSyn7* (−9.0 kcal·mol^-1^) was found in (*R*)-reticuline and sinoacutine; the amino acid residues for hydrophobic interaction between (*R*)-reticuline and *SinSyn7* protein were Phe132, Leu238, Val330, Ala331, and Val400, and the hydrogen bonds formed were Gly131, Gly396, and Val400 (Fig 4B), suggesting that the hydrophobic interaction and hydrogen bonding were the main forces in the binding process of (*R*)-reticuline to *SinSyn7*; the amino acid residues for hydrophobic interaction between sinoacutine and *SinSyn7* were Phe132, Leu238, Val330, Val400, Ile511, and Leu512, and no hydrogen bond was formed (Fig 4C). A high binding free energy to *SinSyn7* (−8.7 kcal·mol^-1^) was found in (*S*)-reticuline; the amino acid residues for hydrophobic interaction were Val400 and Ile511, and the hydrogen bonds formed were Gly131, Ser335, Leu398, and Leu399 (Fig 4D), suggesting that the hydrophobic interaction and hydrogen bonding were the main forces in the binding process of (*S*)-reticuline to *SinSyn7*, and hydrogen bonding was dominant. The highest binding free energy to *SinSyn7* (−7.3 kcal·mol^-1^) was found in (*S*)-scoulerine, with fewer binding sites; the amino acid residues for hydrophobic interaction were Phe132, Leu238, Ile511, and Leu512, and the amino acid residues for hydrogen bonding were Gly131 and Thr327 (Fig 4F).

The *SinSyn7* gene was a homologous gene of sinoacutine synthase. Based on the study by Chen et al., the catalytic substrate of SinSyn was (*S*)-reticuline. Therefore, the binding mode with the highest molecular docking score between *SinSyn7* protein and (*S*)-reticuline was investigated [30]. As shown in (S3 Table), the mutation energy of Gly131, Phe132, Gly396, Val400, and Leu512 was greater than 0.5 after mutation to alanine, which led to decreased affinity between enzyme and substrate; therefore, it can be inferred that sites 131, 132, 396, 400, and 512 were the key amino acid residues for the (*S*)-reticuline reactions catalyzed by *SinSyn7*.

**Fig 5:**
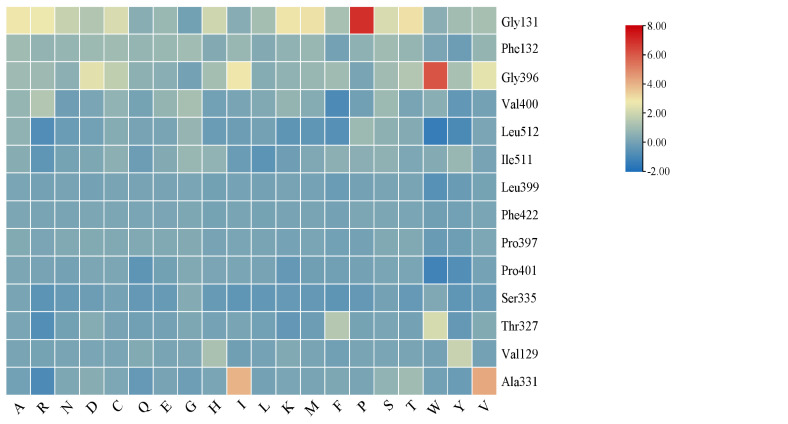
Heat map of the difference in the binding free energy to substrate between mutant and wild type. Red: ΔΔG > 0; yellow: ΔΔG = 0, blue: ΔΔG < 0.

A total of 280 results were obtained by saturation mutation scan of Gly131, Phe132, Gly396, Val400, and Leu512, as well as Ala331, Ile511, Leu399, Val400, and Pro397, which were possible activity centers for molecular docking (**Fig 5**).

As shown in Fig 5, the color in the heat map of Gly131 mutation to Pro and Gly396 mutation to Trp was red, which suggested increased binding free energy compared with the wild type, resulting in decreased affinity between enzyme and substrate; therefore, Gly131 was considered not suitable for mutation to Pro, and Gly396 was considered not suitable for mutation to Trp. When Gly131 was mutated to other 19 amino acids, yellow was dominant in the heat map, which suggested no significant change in the binding free energy for most of the mutants in Gly131 compared to the wild type, and Gly131 was considered not suitable for mutations. When sites Val400, Leu512, Ile511, Leu399, Pro401, and Ser335 were mutated to other 19 amino acids, the color in the heat map contained blue, which suggested that the mutations decreased the binding free energy, resulting in increased affinity between enzyme and substrate and increased interactions. Therefore, it was speculated that Val400, Leu512, Ile511, Leu399, Pro401, and Ser335 were suitable mutation sites to improve enzyme activity.

In order to investigate specific mutations, the mutation energy in the saturation mutation results was arranged in ascending order. The top 20 results for blue from dark to light in Fig 5 are shown in (S4 Table). The mutation energy of the first 12 mutations (from Leu512 → Trp to Ser335 → Arg) in the table was less than −0.5, which suggested that mutations resulted in increased interactions; therefore, these 12 mutations were considered the best for this virtual screening. The mutation energy of the remaining 7 mutations (from Ser335 → Phe to Ser335 → Leu) in the table ranged from −0.5 to −0.4, suggesting that there may be mutation sites in these mutations to improve catalytic performance; therefore, these mutations can also be included in the mutation screening database.

### Heterologous Expression of *SinSyn7* protein

**Fig 6:**
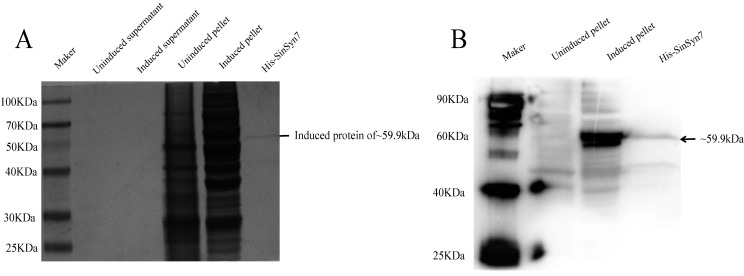
Expression analysis and purification of recombinant *SinSyn7* protein. A. *SinSyn7* protein expression in the uninduced supernatant, induced supernatant, uninduced pellets, induced pellets, and purified samples was observed by 12.5% SDS-PAGE with Coomassie blue staining; B. *SinSyn7* protein expression in the uninduced pellets, induced pellets, and purified induced pellets as validated by Western blotting with anti-His antibody.

The pESC-His-*SinSyn7* vector was introduced into WAT11, and the WAT11 that failed in induced expression (pESC-His-*SinSyn7*) was used as a control. SDS-PAGE was used to analyze whether there was expressed recombinant *SinSyn7* protein in the proteins separated from the uninduced pellets, induced pellets, uninduced supernatant, and induced supernatant. The analysis results showed that there were no bands around 59.9 kDa in the uninduced supernatant, induced supernatant, or uninduced pellets, and expressed recombinant protein (59.9 kDa) was found in the induced pellets and purified samples (Fig 6A). In addition, the anti-His antibody was subjected to Western blotting, and the results showed that the recombinant *SinSyn7* protein with a histidine tag was validated in the induced pellet sample (Fig 6B). It can be seen that the *SinSyn7* gene could be expressed heterologously and intracellularly in WAT11.

### HPLC results of sinoacutine content

The linear regression equation obtained based on the sinoacutine reference standard was y = 118488x − 2000000 and r = 0.9906, indicating good linearity for sinoacutine at a concentration range of 10–200 μg·mL^-1^. The sinoacutine content in the roots, stems, and leaves of different *Sinomenium acutum* plants calculated based on the standard curve is shown in (S5 Table). Based on the results of sinoacutine content in different tissues of the same plant, the rankings of tissues by sinoacutine content were roots > stems > leaves in the 4 selected plants. Based on the results of sinoacutine content in the same tissue of different plants, compared with No. 2, No. 28, and No. 33 plants, No. 12 plant exhibited the highest sinoacutine content, with the sinoacutine content in the roots, stems, and leaves being 55.98 μg·mL^-1^, 26.10 μg·mL^-1^ and 19.65 μg·mL^-1^, respectively.

### Tissue-specific expression results of *SinSyn7* gene

**Fig 7:**
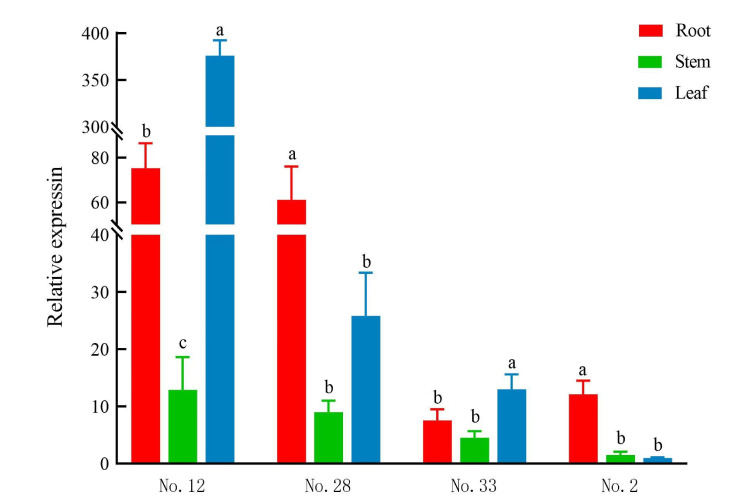
Relative expression of *SinSyn7* gene in different *Sinomenium acutum* plants and different tissues.

The relative expression of *SinSyn7* gene in different tissues of Sinomenium acutum plants is shown in (Fig 7). In the No. 2 plant, the expression of the *SinSyn7* gene was the highest in roots and the lowest in leaves. In No. 28 plant, the expression of the *SinSyn7* gene was the highest in roots and the lowest in stems. In No. 12 and No. 33 plants, the expression of the *SinSyn7* gene was the highest in leaves and the lowest in stems. The expression in roots was always greater than that in stems. The deviation of expression in leaves may be due to the age and tenderness of leaves. Based on the results of *SinSyn7* gene expression in the same tissue of different plants, the ranking of plants by *SinSyn7* gene expression in roots in descending order was No. 12, No. 28, No. 2, and No. 33 plants, that in stems in descending order was No. 12, No. 28, No. 33, and No. 2 plants, and that in leaves in descending order was No. 12, No. 28, No. 33, and No. 2 plants. In general, the expression of the *SinSyn7* gene was the highest in the No. 12 plant, followed by the No. 28 plant, and the expression was the lowest in the No. 2 plant. The results suggested that the relative expression of the SinSyn7 gene varied in different plants and different tissues.

### Analysis of correlation of sinoacutine content with *SinSyn7* gene expression in different tissues of *Sinomenium acutum*

Pearson correlation analysis showed that the correlation coefficient between the *SinSyn7* gene expression and the sinoacutine content in the roots, stems, and leaves of *Sinomenium acutum* was −0.213, which suggested a negative and insignificant correlation (*P* = 0.507) (S6 Table). The results suggested that there was no significant correlation between the expression of the *SinSyn7* gene and the production of sinoacutine in different tissues.

## Discussion

The sinoacutine synthetase *SinSyn7* gene is homologous to the key enzyme responsible for catalyzing sinoacutine synthesis from (S)-reticuline, playing a pivotal role in deciphering the complete sinomenine biosynthetic pathway. In this study, the complete CDS of the *SinSyn7* gene was obtained by the combination of RACE and complete genome data. The bioinformatics analysis showed that *SinSyn7* protein was hydrophilic without signal peptide, with a molecular weight of 59.9 kDa, 523 amino acids, a theoretical isoelectric point PI of 7.28; *SinSyn7* protein had a transmembrane domain, and belonged to the cytochrome P450 superfamily, and α-helixes and random coils were dominant in the secondary structure. *SinSyn7* protein was mainly founded in chloroplasts, and was closely related to *Papaver somniferum* synthetase.

It is important to analyze the binding energy and binding modes of proteins and ligands through molecular docking to investigate the possible catalytic functions and functional differences of enzymes [31]. Singh et al. found that molecular modeling and docking of recombinant HAP-phytase of a thermophilic mold revealed insights into molecular catalysis and biochemical properties [32]. In this study, a homologous model of the *SinSyn7* protein was constructed. Using the model constructed by AlphaFold2 for molecular docking, the binding free energy of the 6 ligands to *SinSyn7* was between −9.0 and −7.3 kcal·mol^-1^. Among them, the lowest binding free energy to *SinSyn7* protein (−9.7 kcal·mol^-1^) was found in corytuberine, followed by (*R*)-reticuline and sinoacutine (−9.0 kcal·mol^-1^), and then (*S*)-reticuline (− 8.7 kcal·mol^-1^); the highest binding free energy to *SinSyn7* (−7.3 kcal·mol^-1^) was found in (*S*)-scoulerine. According to literature, the molecular docking score was less than −7.0 kcal·mol^-1^; the target had a strong binding ability to the compound, with the score being less than −5.0 kcal·mol^-1^ and greater than −7.0 kcal·mol^-1^; the compound small molecule had a good binding ability to the target, with a score between −5.0 kcal·mol^-1^ and −4.25 kcal·mol^-1^, and the compound had a certain binding ability to the target [33]. The results showed that the 6 ligands exhibited strong binding ability, among which corytuberine had the strongest binding ability, followed by (*R*)-reticuline and sinoacutine, and then (*S*)-reticuline, and (*S*)-scoulerine had the worst binding ability. Therefore, it can be speculated that *SinSyn7* protein may be able to catalyze the substrates (*S*)-reticuline and (*R)*-reticuline to produce sinoacutine, corytuberine, (*R)*-scoulerine, and (*S*)-scoulerine, which can be validated by subsequent enzyme catalytic experiments. In the virtual mutation study, the alanine mutation scanning library showed that sites 131, 132, 396, 400, and 512 were the key amino acid residues for the (*S*)-reticuline reactions catalyzed by *SinSyn7* based on the constructed saturation mutagenesis library, Val400, Leu512, Ile511, Leu399, Pro401, and Ser335 were considered as suitable mutation sites to improve enzyme activity, and 12 mutants from Leu512 → Trp to Ser335 → Arg could improve substrate affinity, which may provide theoretical support for subsequent modification of *SinSyn7* enzyme and provide a reference for *SinSyn7* enzyme with high activity and single product.

For the first time, the *SinSyn7* gene was obtained from *Sinomenium acutum* and was expressed in a bacterial system (*Saccharomyces cerevisiae* WAT11). The molecular weight of the galactose-induced recombinant *SinSyn7* protein observed in the pellets was approx. 59.9 kDa, and the purification effect of the induced expressed *SinSyn7* enzyme was good. Interestingly, no recombinant protein was detected in the supernatant, which may be due to the fact that the recombinant *SinSyn7* protein was an intracellularly expressed protein rather than a secreted protein, and there was indeed no signal peptide. The *SinSyn7* enzyme was validated using an anti-His antibody. Bioinformatics analysis showed that *SinSyn7* was a membrane protein; however, due to low protein yield and high instability of membrane proteins and considering that the structural biology of membrane proteins is difficult for research, the transmembrane domain of *SinSyn7* gene was removed to facilitate the study [34]. The purified *SinSyn7* protein can be used for subsequent polishing andlays a foundation for the subsequent determination of enzyme activity and the acquisition of enzyme crystal structure.

The results of this study showed that the ranking of different tissues of the same plant by sinoacutine content in descending order was roots, stems, and leaves. Xie et al.‘s study demonstrated a comparable trend for sinomenine content, with the highest concentration in roots, followed by stems, and the lowest in leaves [35], indicating the trend of sinomenine content in different tissues of *Sinomenium acutum* plants was the same as that of sinoacutine content, i.e., roots > stems > leaves. The gene expression results showed that the *SinSyn7* gene was expressed extensively in *Sinomenium acutum* plants, with higher expression in roots and stems. Out of the 4 selected plants, the expression of the *SinSyn7* gene in the roots, stems, and leaves of the No. 12 plant was the highest, which was consistent with the results of sinoacutine content, that is, the sinoacutine content in the roots, stems, and leaves of No. 12 plant was the highest. Although the correlation analysis showed that there was no significant correlation between the *SinSyn7* expression and the sinoacutine content in different tissues, a certain correlation between the two can also be considered. The study by Zeng et al. showed that the expression of alkaloid synthase genes in the *Sinomenium acutum* plants used for transcriptome sequencing was positively correlated with the sinomenine content [10]. As a result, a certain correlation between the sinomenine content and the *SinSyn7* expression can be considered when investigating the same tissue of different plants. Therefore, the No. 12 plant could be selected as the material for further functional research to obtain the CDS of *SinSyn7*, so as to provide a reference for subsequent functional determination of *SinSyn7.* This study provides a theoretical basis for the screening of high-quality sinoacutine synthetase, and lays a foundation for the study of sinomenine biosynthesis pathway.

This study offers initial insights into the biological properties and potential functions of the *SinSyn7* gene, yet several limitations should be acknowledged. First, the virtual mutation analysis via molecular docking identified five key amino acid sites and 12 single mutations with enhanced binding capacity. However, the accuracy of these predictions hinges on the structural homology of template proteins and the selection of force-field parameters. Verification through in vitro site-directed mutagenesis and enzyme activity assays is essential. Second, the intracellular soluble form of the *SinSyn7* protein obtained from the heterologous expression system might diverge from its natural state due to the absence of plant-specific post-translational modifications or cofactors, potentially affecting its catalytic properties. Third, while a correlation exists between gene expression patterns and sinoacutine content across different tissues, the role of *SinSyn7* in regulating target alkaloid synthesis has not been directly confirmed through gene silencing or overexpression experiments. Fourth, although the study has focused on the two substrates, *(S)*- and *(R)*-reticuline, the enzyme’s involvement in catalyzing other intermediates remains unexplored.

To address these limitations, several research directions are proposed:

(1) Utilize CRISPR/Cas9 gene editing or RNA interference to generate *SinSyn7* loss -of-function plants. Metabolomic analysis can then clarify its role in sinoacutine and other alkaloid synthesis.
(2) Recombinantly express the 12 single mutants identified through virtual screening for in vitro validation of their substrate binding and catalytic efficiency.
(3) Optimize the expression system by employing eukaryotic systems such as Saccharomyces cerevisiae or plant suspension cells to enhance protein solubility. Additionally, solve the crystal structure of *SinSyn7* to elucidate its catalytic mechanism.
(4) Apply molecular dynamics simulations and quantum chemical calculations to investigate the roles of key amino acid residues in substrate recognition and stereoselective catalysis.
(5) Examine the synergistic interactions of *SinSyn7* with other P450 enzymes or transporter proteins, and construct a model for its regulation within plant secondary metabolic pathways.
(6) Develop engineered strains or plant chassis using synthetic biology approaches to achieve efficient heterologous synthesis of target alkaloids.

These steps will help overcome the current limitations and advance the understanding and application of the *SinSyn7* gene.

## Conclusion

*SinSyn7* was a CDS sequence obtained by using RACE, with a length of 1620 bp and encoding 523 amino acids. This gene belonged to the cytochrome P450 superfamily and encoded a hydrophilic transmembrane protein. This protein was a subcellular protein located in chloroplasts and was closely related to Papaver somniferum synthetase. *SinSyn7*-encoded protease may be involved in the production of sinoacutine, corytuberine, (*R*)-scoulerine, and (*S*)-scoulerine by the catalytic substrates (*S*)-reticuline and (*R*)-reticuline. In the virtual mutation study, a total of 5 sites were found to be the key amino acid residues for the (*S*)-reticuline reactions catalyzed by *SinSyn7*, and at least 12 groups of single mutations could enhance the binding affinity of *SinSyn7* protein to (*S*)-reticuline. The heterologously expressed recombinant *SinSyn7* protein was an intracellularly expressed protein rather than a secreted protein. The purified *SinSyn7* protein can be used for subsequent polishing, and also lays a foundation for the subsequent determination of enzyme activity and the acquisition of enzyme crystal structure. *SinSyn7* was well expressed in various tissues of *Sinomenium acutum*, and was most expressed in roots and stems. Based on the changes in the sinoacutine content in different tissues, it was speculated that *SinSyn7* may be involved in regulating the production and accumulation of sinoacutine in different tissues through tissue expression specificity, which is still subject to further experimental validation.

## Supporting information

S1 Table Primers used in this study. (DOCX)

S2 Table Molecular docking of *SinSyn7* with ligands. Val: valine; Ile: isoleucine; Phe: phenylalanine; Leu: leucine; Ala: alanine; Gly: glycine; Ser: serine; Thr: threonine.
(DOCX)

S3 Table Alanine scan. (DOCX)

S4 Table Saturation mutation scan. (DOCX)

S5 Table Determination results of sinoacutine content. (DOCX)

S6 Table Correlation between the sinoacutine content and *SinSyn7* gene expression in different tissues. (DOCX)

S1 File Original Blots for Fig 1. (PDF)

S2 File Original Blots for Fig 6. (PDF)
