# Supplementary material for: Cloning, heterologous expression, and expression analysis of SinSyn7 gene from Sinomenium acutum
Source: PLoS One. 2025 Jul 9;20(7):e0327959. doi: 10.1371/journal.pone.0327959 (PMC12240356; doi:10.1371/journal.pone.0327959)
Supplement: S1 Table — (DOCX) [file pone.0327959.s001.docx]

**S 1 Table. Primers used in this study.**

| **Primer name** | **Primer Sequence (5’→3’)** | **Note** |
| --- | --- | --- |
| GSP 1 | ATTGGAATCGTCACCCGTTTCAGCTG | 5’RACE |
| GSP 2 | GCCATGCTATCAACCCCAGCAACTAG | 5’RACE |
| GSP3 | AGTAGCCAGCCACGTGACAATCCTC | 5’RACE |
| M13-F | TGTAAAACGACGGCCAGT | Colony PCR |
| M13-R | CAGGAAACAGCTATGACC | Colony PCR |
| SinSyn7-F | CCATGCGAAACACTCAGCAG | qPCR |
| SinSyn7-R | CATTGACACCAGAACGTCGAG | qPCR |
