# Supplementary material for: Cloning, heterologous expression, and expression analysis of SinSyn7 gene from Sinomenium acutum
Source: PLoS One. 2025 Jul 9;20(7):e0327959. doi: 10.1371/journal.pone.0327959 (PMC12240356; doi:10.1371/journal.pone.0327959)
Supplement: S2 Table — Val: valine; Ile: isoleucine; Phe: phenylalanine; Leu: leucine; Ala: alanine; Gly: glycine; Ser: serine; Thr: threonine. (DOCX) [file pone.0327959.s002.docx]

**S 2 Table. Molecular docking of SinSyn7 with ligands.**

| **Ligand** | **CAS Accession No.** | **Binging Energy (kcal·mol^-1^)** | **Hydrophobic Effect** | **Hydrogen Bond** |
| --- | --- | --- | --- | --- |
| (S)-reticuline | 1699-46-3 | −8.7 | Val400, Ile511 | Gly131, Ser335, Leu398, Leu399 |
| (R)-reticuline | 3968-19-2 | −9.0 | Phe132, Leu238, Val330, Ala331, Val400 | Gly131, Gly396, Val400 |
| Sinoacutine | 4090-18-0 | −9.0 | Phe132, Leu238, Val330, Val400, Ile511, Leu512 | - |
| Corytuberine | 517-56-6 | −9.7 | Phe132, Val330, Val400, Ile511 | - |
| (R)-scoulerine | 6451-72-5 | −8.0 | Phe132, Leu238, Val330, Ala331, Val400, Ile511, Leu512 | Gly131, Ile511 |
| (S)-scoulerine | 6451-73-6 | −7.3 | Phe132, Leu238, Ile511, Leu512 | Gly131, Thr327 |

Val: valine; Ile: isoleucine; Phe: phenylalanine; Leu: leucine; Ala: alanine; Gly: glycine; Ser: serine; Thr: threonine.
