# Supplementary material for: Cloning, heterologous expression, and expression analysis of SinSyn7 gene from Sinomenium acutum
Source: PLoS One. 2025 Jul 9;20(7):e0327959. doi: 10.1371/journal.pone.0327959 (PMC12240356; doi:10.1371/journal.pone.0327959)
Supplement: S3 Table — (DOCX) [file pone.0327959.s003.docx]

**S 3 Table. Alanine scan.**

| **Amino Acid Mutation** | **Mutation Energy (kcal·mol^-1^)** | **Mutagenesis** |
| --- | --- | --- |
| Gly131→Ala | 2.86 | Destabilizing |
| Phe132→Ala | 0.97 | Destabilizing |
| Gly396→Ala | 0.95 | Destabilizing |
| Val400→Ala | 0.74 | Destabilizing |
| Leu512→Ala | 0.65 | Destabilizing |
| Ile511→Ala | 0.43 | Neutral |
| Leu238→Ala | 0.39 | Neutral |
| Pro397→Ala | 0.31 | Neutral |
| Arg114→Ala | 0.22 | Neutral |
| Pro401→Ala | 0.19 | Neutral |
