# Supplementary material for: Cloning, heterologous expression, and expression analysis of SinSyn7 gene from Sinomenium acutum
Source: PLoS One. 2025 Jul 9;20(7):e0327959. doi: 10.1371/journal.pone.0327959 (PMC12240356; doi:10.1371/journal.pone.0327959)
Supplement: S4 Table — (DOCX) [file pone.0327959.s004.docx]

**S 4 Table. Saturation mutation scan.**

| **Amino Acid Mutation** | **Mutation Energy (kcal·mol^-1^)** | **Mutagenesis** |
| --- | --- | --- |
| Leu512→Trp | −1.39 | Stabilizing |
| Pro401→Trp | −1.16 | Stabilizing |
| Leu512→Tyr | −0.91 | Stabilizing |
| Ala331→Arg | −0.89 | Stabilizing |
| Val400→Phe | −0.88 | Stabilizing |
| Leu512→Arg | −0.8 | Stabilizing |
| Thr327→Arg | −0.77 | Stabilizing |
| Pro401→Tyr | −0.74 | Stabilizing |
| Leu512→Phe | −0.68 | Stabilizing |
| Leu399→Trp | −0.65 | Stabilizing |
| Ile511→Leu | −0.54 | Stabilizing |
| Ser335→Arg | −0.54 | Stabilizing |
| Ser335→Phe | −0.5 | Neutral |
| Leu512→Lys | −0.49 | Neutral |
| Pro401→Gln | −0.49 | Neutral |
| Ile511→Arg | −0.48 | Neutral |
| Leu512→Met | −0.47 | Neutral |
| Ser335→Tyr | −0.46 | Neutral |
| Ser335→Ile | −0.45 | Neutral |
| Ser335→Leu | −0.4 | Neutral |
