# Supplementary material for: Cloning, heterologous expression, and expression analysis of SinSyn7 gene from Sinomenium acutum
Source: PLoS One. 2025 Jul 9;20(7):e0327959. doi: 10.1371/journal.pone.0327959 (PMC12240356; doi:10.1371/journal.pone.0327959)
Supplement: S5 Table — (DOCX) [file pone.0327959.s005.docx]

**S 5 Table. Determination results of sinoacutine content.**

| **Plant** | **Tissue** | **Sinoacutine Content (μg·mL^-1^)** |
| --- | --- | --- |
| No. 2 | Root | 36.84 |
|  | Stem | 18.50 |
|  | Leaf | 17.33 |
| No. 33 | Root | 45.12 |
|  | Stem | 18.68 |
|  | Leaf | 17.52 |
| No. 28 | Root | 33.84 |
|  | Stem | 18.96 |
|  | Leaf | 17.45 |
| No. 12 | Root | 55.98 |
|  | Stem | 26.10 |
|  | Leaf | 19.65 |
