# Supplementary material for: Cloning, heterologous expression, and expression analysis of SinSyn7 gene from Sinomenium acutum
Source: PLoS One. 2025 Jul 9;20(7):e0327959. doi: 10.1371/journal.pone.0327959 (PMC12240356; doi:10.1371/journal.pone.0327959)
Supplement: S6 Table — (DOCX) [file pone.0327959.s006.docx]

**S 6 Table. Correlation between the sinoacutine content and *SinSyn7* gene expression in different tissues.**

|  | **Sinoacutine Content** | **Gene Expression** |
| --- | --- | --- |
| Sinoacutine Content | 1 | −0.213 |
|  | - | 0.507 |
| Gene Expression | −0.213 | 1 |
|  | 0.507 | - |
