# Supplementary figures and images for: Cloning, heterologous expression, and expression analysis of SinSyn7 gene from Sinomenium acutum
Source: PLoS One. 2025 Jul 9;20(7):e0327959. doi: 10.1371/journal.pone.0327959 (PMC12240356; doi:10.1371/journal.pone.0327959)

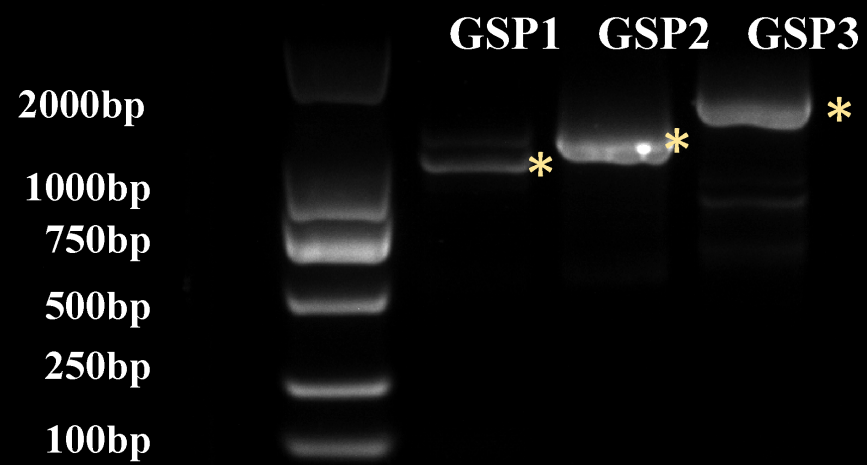

2000bp  
1000bp  
750bp  
500bp  
250bp  
100bp

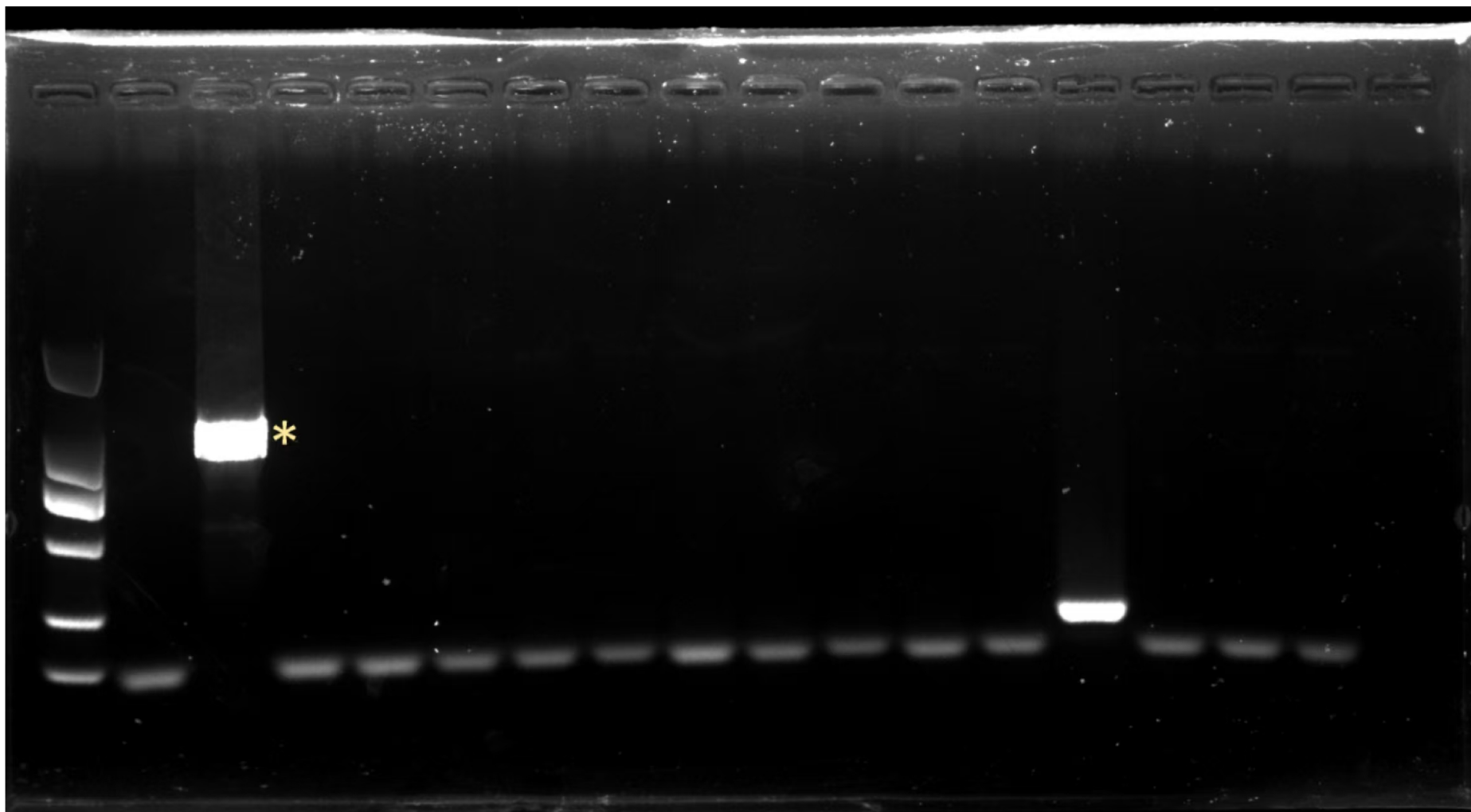

Supplement: S1 File — (PDF) [file pone.0327959.s007.pdf]

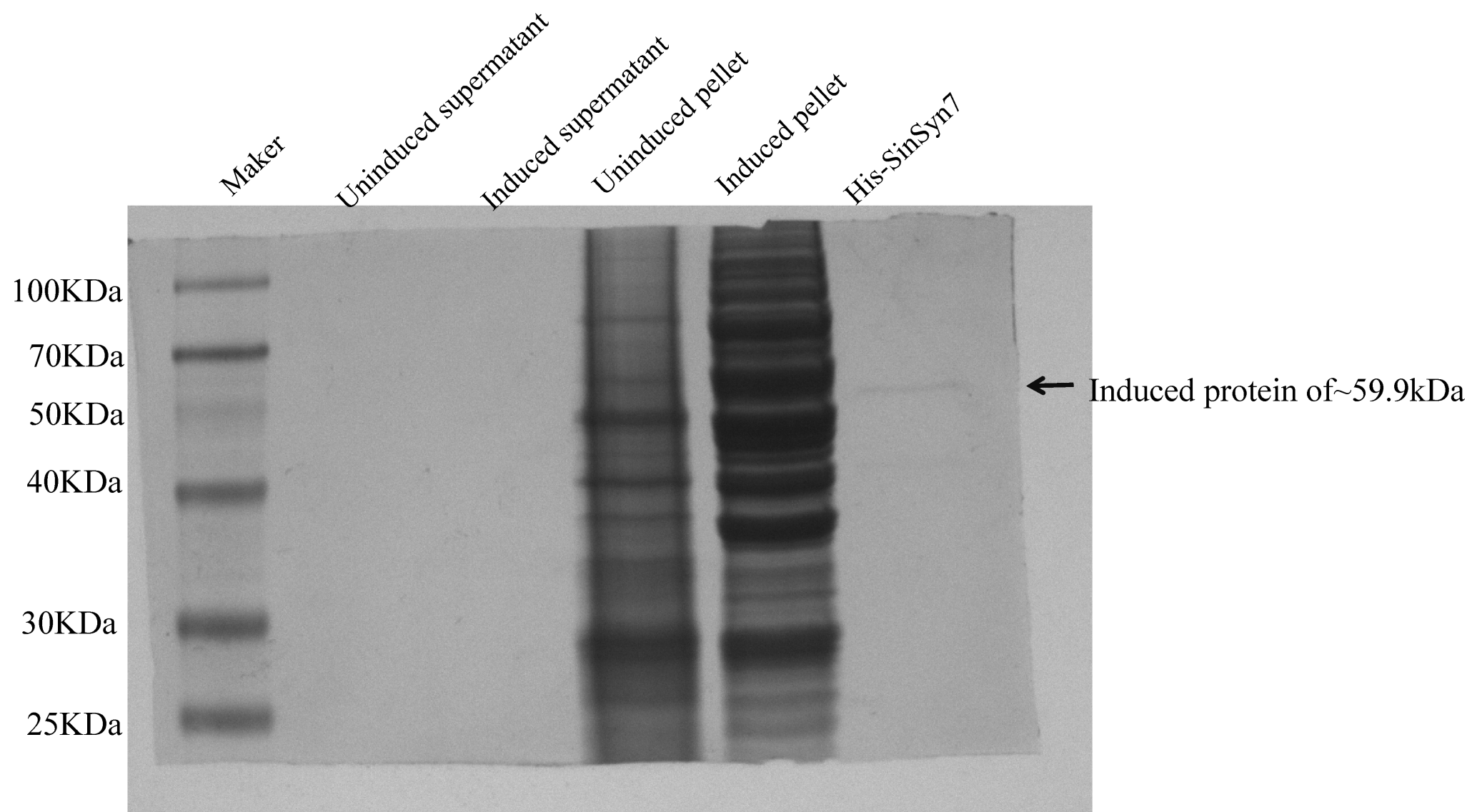

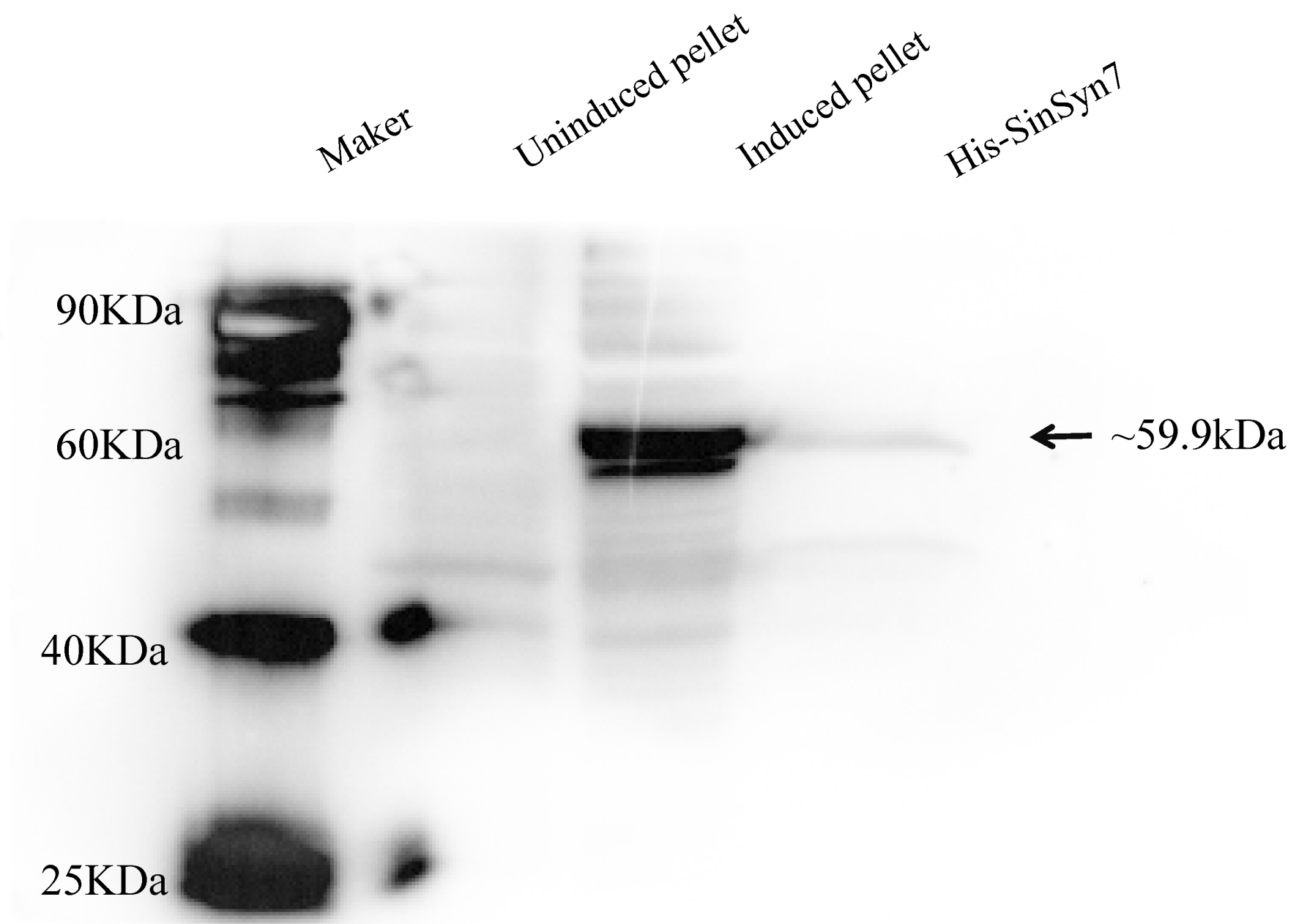

Supplement: S2 File — (PDF) [file pone.0327959.s008.pdf]
